# Mutational Signature Changes in Patients With Metastatic Squamous Cell Carcinoma of the Anal Canal

**DOI:** 10.1093/oncolo/oyad326

**Published:** 2023-12-16

**Authors:** Michael LaPelusa, Christopher Cann, Kristen K Ciombor, Cathy Eng

**Affiliations:** Department of Internal Medicine, Vanderbilt University Medical Center, Nashville, TN, USA; Division of Hematology and Oncology, Department of Internal Medicine, Vanderbilt-Ingram Cancer Center, Vanderbilt University Medical Center, Nashville, TN, USA; Division of Hematology and Oncology, Department of Internal Medicine, Vanderbilt-Ingram Cancer Center, Vanderbilt University Medical Center, Nashville, TN, USA; Division of Hematology and Oncology, Department of Internal Medicine, Vanderbilt-Ingram Cancer Center, Vanderbilt University Medical Center, Nashville, TN, USA

**Keywords:** squamous cell carcinoma of the anal canal, anal cancer, circulating tumor DNA, next generation sequencing

## Abstract

**Purpose:**

We examined the concordance of genetic mutations between pretreatment tumor tissue and posttreatment circulating tumor DNA (ctDNA) in patients with metastatic squamous cell carcinoma of the anal canal (SCCA) and assessed the impact of therapy on this concordance.

**Methods:**

We analyzed next-generation sequencing reports from pretreatment tumor tissue and posttreatment ctDNA in 11 patients with metastatic SCCA treated at Vanderbilt University Medical Center between 2017 and 2021.

**Results:**

Among the mutations identified in posttreatment ctDNA, 34.5% were also found in pretreatment tumor tissue, while 47.6% of pretreatment tumor tissue mutations were found in posttreatment ctDNA. Four patients had preservation of potentially actionable mutations in both pretreatment tissue and posttreatment ctDNA, while 7 patients had newly identified mutations in posttreatment ctDNA that were not present in pretreatment tumor tissue.

**Conclusion:**

Patients with SCCA demonstrate a high degree of temporal mutational heterogeneity. This supports the hypothesis that ctDNA can serve as a real-time tracking mechanism for solid tumors’ molecular evolution in response to therapy. Our findings highlight the potential of ctDNA in identifying emerging actionable mutations, supplementing information from tissue-based genomic assessments. Further research, ideally with larger and multi-institutional cohorts, is needed to validate our findings in this relatively rare tumor type.

## Teaser

This study aimed to describe how tissue-based and ctDNA-based mutational concordance, as identified by next-generation sequencing, is affected by chemoradiation and systemic therapy in the context of patients’ unique clinicodemographic features.

**Implications for Practice**

The mutational profile in tumors of patients with anal cancer changes in response to treatment. Circulating tumor DNA may be used to identify new mutations that could be acted upon with targeted therapy when patients’ cancer progresses on chemotherapy and immunotherapy.

## Introduction

In 2023, there will be an estimated 9760 patients diagnosed with anal cancer and 1870 deaths attributable to anal cancer in the US.^1^ The incidence of squamous cell carcinoma of the anal canal (SCCA) in the US increased by 2.7% per year, and anal cancer–related deaths increased by 3.1% per year, from 2001 to 2015.^2^ Several factors that increase the risk of developing SCCA have been identified, including more than 10 sexual partners, receptive anal intercourse before the age of 30, chronic immunosuppressed states such as solid-organ transplant or infection with human immunodeficiency virus, active tobacco use, and being older than 50 years old.^3,4^ Further, human papillomavirus (HPV) infection is associated with up to 95% of cases of SCCA.^5^ Chemoradiation is typically administered to patients with SCCA with locoregional disease, while patients with metastatic disease are treated with combination chemotherapy.^6-10^ Immunotherapy has also shown efficacy in patients with refractory metastatic disease and is being studied, in combination with chemotherapy for use in the first-line setting for patients with metastatic disease.^11,12^ Achieving disease control in patients with metastatic SCCA is a challenge, with one analysis finding that patients with metastatic SCCA treated with combination chemotherapy experience a median progression-free survival of 7 months and median overall survival of 22 months.^8^

Circulating tumor DNA (ctDNA) has been used effectively, in conjunction with imaging, to measure therapeutic response to chemoradiation in patients with locoregional anal cancer.^13^ Detection of HPV ctDNA is an emerging tool to detect minimal residual disease in patients with low disease burden, particularly following chemoradiation, and has been shown to be a mechanism to identify patients at high risk of recurrence in this setting.^14^

**Figure 1.**
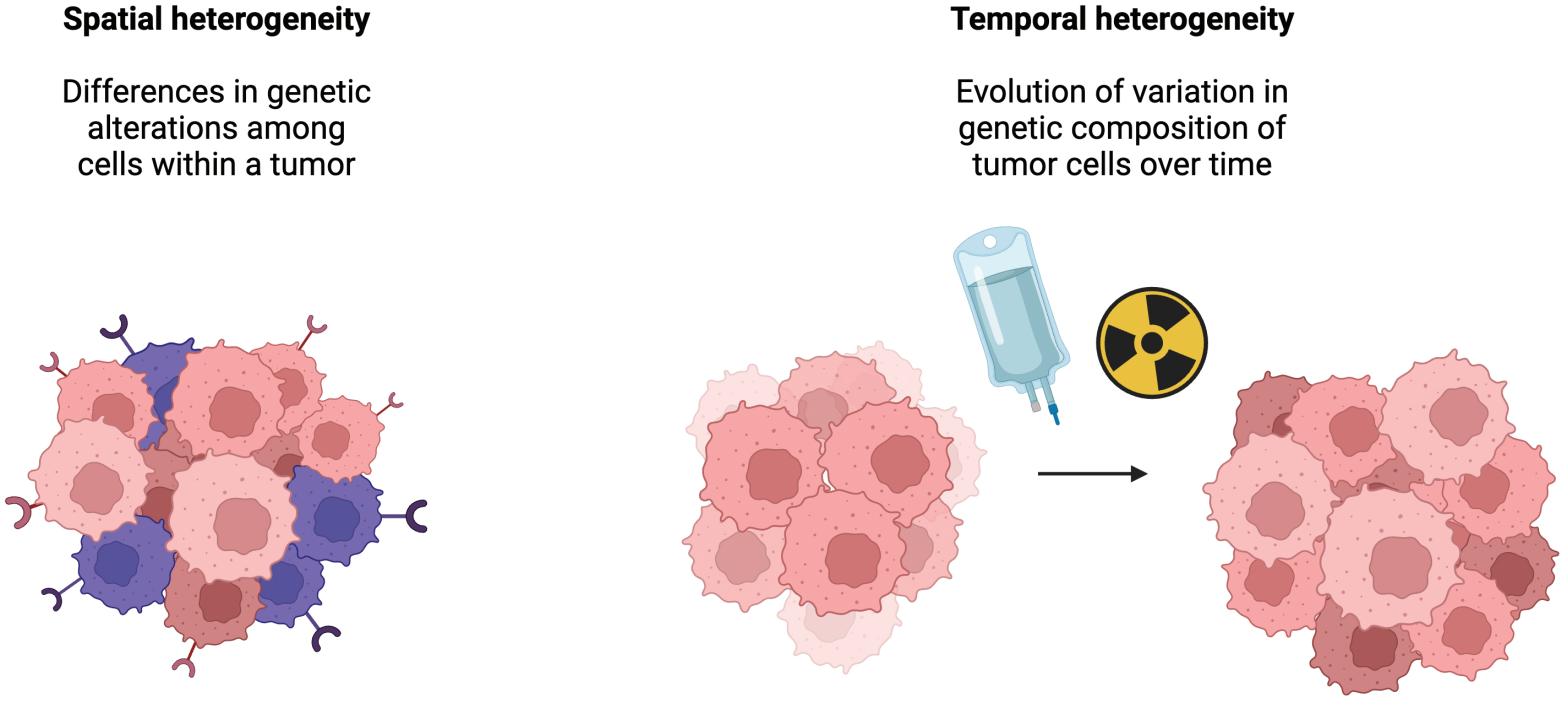
Spatial and temporal tumor heterogeneity.

However, ctDNA has not been well studied in patients with SCCA as a means to understand spatial mutational heterogeneity (within a tumor or across sites of disease within one patient). This heterogeneity can lead to incomplete genomic information obtained via tumor biopsy.^15,16^ Temporal mutational heterogeneity, or the evolution of variation in the genetic composition of tumor cells over time that can occur as a response to treatment and ultimately leads to resistance to a particular therapy, represents another challenge to cancer treatment.^16^ A way to confront these challenges may be to serially assess a tumor’s mutational profile dynamically during treatment with peripheral blood measurements of ctDNA, particularly when patients exhibit disease progression, to identify newly acquired mutations that could serve as a target for therapy and allow for personalized changes in treatment while forgoing an invasive tissue biopsy (Fig. 1).

The relatively low incidence and prevalence of SCCA pose a unique challenge to characterizing detailed tumoral genomic information in this patient population. Few analyses of the genomic profile of metastatic SCCA have been performed.^17,18^ The concordance, or lack thereof, of genomic alterations between tumor tissue and ctDNA has been reported in many tumor types, including *EGFR* in non-small cell lung cancer (NSCLC), *KRAS* in pancreatic cancer and metastatic colorectal cancer, and *BRAF V600E* in melanoma, but not in SCCA.^19-23^ In this study, we sought to describe how tissue-based and ctDNA-based mutational concordance, as identified by next-generation sequencing (NGS), is impacted by chemoradiation and systemic therapy in the context of patients’ unique clinicodemographic features.

## Methods

We identified 11 patients with histologically confirmed metastatic SCCA treated at Vanderbilt University Medical Center between 2017 and 2021 with tissue-based TEMPUS 648 gene panel reports and ctDNA-based 105 gene panel reports available for analysis. Clinicodemographic features included in our study were: HPV status, clinical stage, the time interval between tissue biopsy and ctDNA collection, the type and amount of therapy administered during the interval between tumor tissue and ctDNA collection, and the mutations identified in each sample. Mutations were classified into predetermined categories on tissue-based TEMPUS 648 gene panel reports and ctDNA-based TEMPUS 105 gene panel reports (“Somatic—potentially actionable,” “Somatic—biologically relevant,” and “Variants of unknown significance”). We reported variant allele frequencies (VAF) if they were included in the sample’s reports.

## Results

Table 1 displays by patient, the source of tissue, HPV status, clinical stage at the time of sample collection, collection Interval, interval therapy, and genes in which mutations were identified. Table 2 displays the same information but includes the specific mutations and genetic aberrations identified by NGS. Eight patients had tissue obtained from their primary tumor in the anal canal, while 3 patients had tissue obtained from sites of metastasis (lung [patient 4], pelvic lymph node [patient 5], and brain [patient 6]). Nine patients were HPV positive, as indicated by p16+ status. Four (1, 2, 8, and 10) patients with mutations in tumor tissue categorized as “somatic—potentially actionable” had the same mutations in ctDNA. In 3 (1, 2, and 10) of these 4 patients, the VAF was lower in ctDNA than in tumor tissue. Two^1,10^ patients with mutations in tumor tissue categorized as “somatic—biologically relevant” had the same mutations in ctDNA. In one of these patients, the VAF was lower in ctDNA than in tumor tissue. Two patients with mutations in tumor tissue categorized as “variants of unknown significance” had the same mutations in ctDNA. In one^2^ of the 3 patients, the VAF was lower in ctDNA than in tumor tissue.

**Table 1.** Clinical and molecular characteristics (abbreviated).

Patient	Sample	HPV status	Stage at Time of Sample Collection	Collection interval (days)	Interval Therapy (cycles)	Somatic—potentially actionable (VAF), if reported)	Somatic—biologically relevant (VAF, if reported)	Variants of unknown significance (VAF, if reported)
1	Anus	Negative	IIA (T2N0M0)	T	-5-FU + mitomycin-C + radiation -carboplatin + paclitaxel (6) -nivolumab (3)	**-CDKN2A (47.4%)** -**TP53 (45.9%)** -CCND1	-**TERT (38.5%)** -FGF19* -FGF3* -FGF4*	-MTHFR (53.1%)* -WEE1 (34.5%)* -CBLB (7.9%)*
	ctDNA		IV	T + 643		**-CDKN2A (1.4%)** -**TP53 (1.3%)**	-**TERT (3.6%)**	-ATM (0.4%)**
2	Anus	p16+	IIIC (T4N1M0)	T	-5-FU + mitomycin-C + radiation -carboplatin + paclitaxel (4)	-**MAPK1 (15.0%)**	-ARID2 (15.1%)* -FANCA (11.2%)*	-SMAD3 (17.4%)* -**MSH2 (16.9%)** -HIST1H1E (15.8%)* -EPHA2 (15.4%)* -LMNA (14.4%)* -FANCA (11.1%)* -DYNC2H1 (7.3%)* -SETD2 (6.9%)*
	ctDNA		IV	T + 414		-**MAPK1 (7.3%)**	None	-KDR (50.4%)** -SDHA (46.8%)** -**MSH2 (6.9%)**
3	Anus	Negative	IIIA (T2N1M0)	T	-5-FU + mitomycin-C + radiation -carboplatin + paclitaxel + nivolumab (2) -5-FU + cisplatin (3) -irinotecan (1)	None	-FAT1 (22.2%)* -CASP8* -ERCC3* -FHIT* -FOXP1* -LRP1B*	-SYNE1 (22.2%)* -PTPN11 (18.1%)
	ctDNA		IV	T + 484		None	-MYC**	-HNF1A (57.4%)**
4	Lung	p16+	IV	T	-carboplatin + paclitaxel (7) -pembrolizumab (10) -FOLFOX (9) -PEN 866 (9)	-PIK3CA (37.0%)	-CYLD (64.1%)* -CREBBP (21.7%)* -EBF1*	-KIF1B (35.0%)*
	ctDNA		IV	T + 1057		None	None	None
5	Pelvic lymph node	p16+	IV	T	-nivolumab (1) -5-FU + cisplatin (3)	-PIK3CA (18.6%)	-STK11 (38.0%) -BCL11B* -SLIT2*	-**STK11 (36.0%)** -TPM1 (33.7%)* -AMER1 (24.1%)* IV-CTRC (22.3%)* -RAD51B (19.7%)* -ETS2 (18.7%)* -GATA2*
	ctDNA		IV	T + 763		None	None	-**STK11 (0.4%)**
6	Brain	p16+	IV	T	-irinotecan + cetuximab (1)	None	-CYLD (83.4%)*	-SETD2 (84.2%)* -SEC63 (45.6%)* -AGO1 (40.6%)* -ATRX (34.7%)*
	ctDNA		IV	T + 763		None	-SDHA (1.3%)**	-FGFR2 (49.7%)** -MAP2K1 (0.9%)**
7	Anus	p16+	IIIC (T4N1M0)	T	-5-FU + mitomycin-C + radiation -carboplatin + paclitaxel (3) -nivolumab (8)	None	-APC	-APOB (25.1%)* -ROS1 (21.8%) -GRM3 (20.1%)* -MYH11 (20.0%)* -PMS2 (19.4%)
	ctDNA		IV	T + 369		None	None	None
8	Anus	p16+	IIA (T2N0M0)	T	-5-FU + mitomycin-C + radiation -nivolumab (8)-	-**PIK3CA (22.8%)**	-KMT2D (43.5%)* -B2M*	-PHOX2B (85.3%)* -HSD3B1 (38.8%)*
	ctDNA		IV	T + 205		-**PIK3CA (24.5%)**	None	-PBRM1 (85.1%)** -DDR2 (49.9%)** -MYCN (48.6%)** -APC (12.1%)** -SDHA (0.9%)** -CDKN2A (0.2%)**
9	Anus	p16+	IIB (T3N0M0)	T	-5-FU + mitomycin-C + radiation -carboplatin + paclitaxel (6) -pembrolizumab (4) -cisplatin + capecitabine (5)	None	-CYLD (23.0%)*	-UBC (26.1%)* -**RAD51C (24.5%)** -**JAK2 (17.9%)**
	ctDNA		IV	T + 989		None	None	-**RAD51C (40.9%)** -**JAK2 (33.8%)** -MTOR (13.9%)** -PMS2 (5.7%)** -BRCA1 (4.8%)**
10	Anus	p16+	IV	T	-carboplatin + paclitaxel (3) -nivolumab (11) -panitumumab (8) -5-FU + panitumumab (8)	-**PIK3CA (45.8%)** -FBXW7 (14.3%) -CDKN2A	-CDKN2B -SOX2*	-SDHC (18.8%)* -EPHA2 (14.9%)* -ABL1 (13.9%)* -CD70 (12.3%)* -SMARCA1 (11.7%)*
	ctDNA		IV	T + 763		-**PIK3CA (14.9%)**	-FBXW7 (28.7%)**	-ERBB2 (28.2%)**
11	Anus	p16+	IIIC (T3N1M0)	T	-5-FU + mitomycin-C + radiation -carboplatin + paclitaxel (3) -atezolizumab plus bevacizumab (92) -SQZ-PBMC-HPV vaccine	-KMT2C/MLLC (18.8%)* -PIK3CA	-EP300 (32.8%)* -CREBBP (28.4%)* -ASXL1 (7.0%)* -BCL6* -MCL1* -SOX2*	-NSD1 (16.7%)* -LRP1B (13.7%)* -DNM2 (7.1%)* -KLHL6 (6.1%)* -ERBB4 (5.7%)*
	ctDNA		IV	T + 1713		None	None	None

Colored text: Indicates a mutation observed in one patient’s tissue-based TEMPUS 648 gene panel report and ctDNA-based 105 gene panel report.

*Indicates mutations observed in tissue-based TEMPUS 648 gene panel reports that were not tested for in ctDNA-based TEMPUS 105 gene panel reports.

**Indicates mutations observed in ctDNA-based TEMPUS 105 gene panel reports that were not present in the same patient’s tissue-based TEMPUS 648 gene panel reports.

Abbreviation: VAF, variant allele frequency.

**Table 2.** Clinical and molecular characteristics (expanded).

Patient	Sample	HPV status	Stage at Time of Sample Collection	Collection interval (days)	Interval Therapy (cycles)	Somatic—potentially actionable			Somatic—biologically relevant			Variants of unknown significance		
						Gene	Mutation	VAF	Gene	Mutation	VAF	Gene	Mutation	VAF
1	Anus	Negative	IIA (T2N0M0)	T	-5-FU + mitomycin-C + radiation -carboplatin + paclitaxel (6) -nivolumab (3)	**CDKN2A**	**p.r80 SG—LOF**	**47.4%**	**TERT**	**c.-146C > T—PM**	**38.5%**	MTHFR*	c.1462A > G p.I448V MV	53.1%
						**TP53**	**p.P151T MV—LOF**	**45.9%**	FGF19*	CNG		WEE1*	c.107A > T p.E36V MV	34.5%
									FGF3*	CNG				
						CCND1	CNG		FGF4*	CNG		CBLB*	c.2401G > A p.D801N MV	7.9%
	ctDNA		IV	T + 643		**CDKN2A**	**p.r80 SG**	**1.4%**	**TERT**	**c.-146C > T– PM**	**3.6%**	ATM **	c.8495G > A p.R2832H MV	0.4%
						**TP53**	**p.P151T MV—LOF**	**1.3%**						
2	Anus	p16+	IIIC (T4N1M0)	T	-5-FU + mitomycin-C + radiation -carboplatin + paclitaxel (4)	**MAPK1**	**p.E322K SRV—GOF**	**15.0%**	ARID2*	p.R274 SG—LOF	15.1%	SMAD3*	c.733G?A p.G245R MV	17.4%
												**MSH2**	**c.57C > A p.F19L MV**	**16.9%**
												HIST1H1E*	c.343G > A p.E115K MV	15.8%
												EPHA2*	c.274G > C p.E92Q MV	15.4%
									FANCA*	p.W183 SG	11.2%	LMNA*	c.307C > T p.Q103 SG	14.4%
												FANCA*	c.604G > A p.D202N MV	11.1%
												DYNC2H1*	c.2692C > T p.R898 SG	7.3%
												SETD2*	c.1271G > A p.R424Q MV	6.9%
	ctDNA		IV	T + 414		**MAPK1**	**p.E322K SRV—GOF**	**7.3%**	None			-KDR**	c.1379G > T p.W460L MV	50.4%
												SDHA**	c.1663 + 3G > C SRV	46.8%
												**MSH2**	**c.57C > A p.F19L MV**	**6.9%**
3	Anus	Negative	IIIA (T2N1M0)	T	-5-FU + mitomycin-C + radiation -carboplatin + paclitaxel + nivolumab (2) -5-FU + cisplatin (3) -irinotecan (1)	None			FAT1*	p.E1027fs frameshift—LOF	22.2%	SYNE1*	c.718G > A p.E240K MV	22.2%
									CASP8*	CNL				
									ERCC3*	CNL				
									FHIT*	CNL		PTPN11	c.22C > T p.H8Y MV	18.1%
									FOXP1*	CNL				
									LRP1B*	CNL				
	ctDNA		IV	T + 484		None			MYC**	CNG		-HNF1A**	c.1336G > A p.V446M MV	57.4%
4	Lung	p16+	IV	T	-carboplatin + paclitaxel (7) -pembrolizumab (10) -FOLFOX (9) -PEN 866 (9)	PIK3CA	p.E726K MV	37.0%	-CYLD*	p.V487fs frameshift—LOF	64.1%	KIF1B*	c.2980G p.D994N MV	35.0%
									CREBBP*	p.R386 SG—LOF	21.7%			
									EBF1*	CNL		CREBBP*	c.1042C > T p.P3485 MV	25.7%
	ctDNA		IV	T + 1057		None			None			None		
5	Pelvic lymph node	p16+	IV	T	-nivolumab (1) -5-FU + cisplatin (3)	PIK3CA	p.E545K MV	18.6%	STK11	p.Q100 SG—LOF	38.0%	**STK11**	**c.333C > G pI111M MV**	**36.0%**
												TPM1*	c.116G > A p.R39K MV	33.7%
												AMER1*	c.2036G > A p.R679Q MV	24.1%
									BCL11B*	CNL		CTRC*	c.718C > T p.R240W MV	22.3%
												RAD51B*	c.234C > G p.F78L MV	19.7%
									SLIT2*	CNL		ETS2*	c.1466T > A p.I489K MV	18.7%
												GATA2*	c.142T > A p.F481 MV	
	ctDNA		IV	T + 763		None			None			-**STK11**	**c.333C > G p.I111M MV**	**0.4%**
6	Brain	p16+	IV	T	-irinotecan + cetuximab (1)	None			CYLD*	p.Q224 SG—LOF	83.4%	SETD2*	c.6230G > A p.R2077Q MV	84.2%
												SEC63*	c.1666del p.V556fs frameshift	45.6%
												AGO1*	c.431c > G p.A144G MV	40.6%
												ATRX*	c.1718G > C p.G573A MV	34.7%
	ctDNA		IV	T + 763		None			SDHA**	c.456 + 2T > C SRV—LOF	1.3%	FGFR2**	c.1364C > T p.T455M MV	49.7%
												MAP2K1**	c.124C > T p.L42F MV	0.9%
7	Anus	p16+	IIIC (T4N1M0)	T	-5-FU + mitomycin-C + radiation -carboplatin + paclitaxel (3) -nivolumab (8)	None			APC	CNL		APOB*	c.4250C > T p.T1417M MV	25.1%
												ROS1	c.274G > A p.E92K MV	21.8%
												GRM3*	c.1296G > C p.K432N MV	20.1%
												MYH11*	c.5125G > C p.E1709Q MV	20.0%
												PMS2	c.1688G > A p.R563Q MV	19.4%
	ctDNA		IV	T + 369		None			None			None		
8	Anus	p16+	(T2N0M0)	T	-5-FU + mitomycin-C + radiation -nivolumab (8)	**PIK3CA**	**p.E545K MV—GOF**	**22.8%**	KMT2D*	p.E1391 SG—LOF	43.5%	PHOX2B*	c.191C > A p.S64Y MV	85.3%
									B2M*	CNL		HSD3B1*	c.503C > T p.A168V MV	38.8%
	ctDNA		IV	T + 205		**PIK3CA**	**p.E545K MV—GOF**	**24.5%**	None			PBRM1**	c.1409A > G p.Y470C MV	85.1%
												DDR2**	c.749T > A p.V250E MV	49.9%
												MYCN**	c.1090C > T p.P364S MV	48.6%
												APC**	c.3426T > A p.N1142K MV	12.1%
												SDHA**	c.1915C > G p.L639V MV	0.9%
												CDKN2A**	c.319C > T p.R107C MV	0.2%
9	Anus	p16+	IIB (T3N0M0)	T	-5-FU + mitomycin-C + radiation -carboplatin + paclitaxel (6) -pembrolizumab (4) -cisplatin + capecitabine (5)	MUTYH*	c.1187G > A p.S344 SG—LOF		CYLD*	p.S344 SG—LOF	23.0%	UBC*	c.910G > A p.G304R MV	26.1%
												**RAD51C**	**c.772C > T p.R258C MV**	**24.5%**
												**JAK2**	**c.3223G > A p.V1075M MV**	**17.9%**
	ctDNA		IV	T + 989		None			None			**RAD51C**	**c.772C > T p.R258C MV**	**40.9%**
												**JAK2**	**c.3223G > A p.V1075M MV**	**33.8%**
												MTOR**	c.5714 + 1G > C SRV	13.9%
												PMS2**	c.1427G > C p.S476T MV	5.7%
												BRCA1**	c.2029_2030delinsTT p.G677L MV	4.8%
10	Anus	p16+	IV	T	-carboplatin + paclitaxel (3) -nivolumab (11) -panitumumab (8) -5-FU + panitumumab (8)	**PIK3CA**	**p.E545K MV—GOF**	**45.8%**	**FBXW7**	**p.R505G MV—LOF**	**14.3%**	SDHC*	c.485C > T p.S162F MV	18.8%
												EPHA2*	c.1987G > A p.E663K MV	14.9%
						CDKN2A	CNL		CDKN2B*	CNL		ABL1*	c.323G > A p.R108Q MV	13.9%
												CD70*	c.380C > A p.T127N MV	12.3%
									SOX2*	CNG		SMARCA1*	c.514G > C p.E172Q MV	11.7%
	ctDNA		IV	T + 763		**PIK3CA**	**p.E545K MV—GOF**	**14.9%**	**FBXW7**	**p.R505G MV—LOF**	**28.7%**	ERBB2**	c.234G > T p.Q78H MV	28.2%
11	Anus	p16+	IIIC (T3N1M0)	T	-5-FU + mitomycin-C + radiation -carboplatin + paclitaxel (3) -atezolizumab plus bevacizumab (92) -SQZ-PBMC-HPV vaccine	KMT2C*	c.4661-1G > T SRV—LOF	18.8%	EP300*	c.4617 + 1G > T SRV—LOF	32.8%	NSD1*	C2230_2231 delinSGT p.S744V MV	16.7%
						PIK3CA	CNG		CREBBP*	p.S1074 SG—LOF	28.4%	LRP1B*	c.13343T > C p.V448A MV	13.7%
									ASXL1*	p.G646fs frameshift—LOF	7.0%	DNM2*	c.1782-5del SRV	7.1%
						FH*	c.1431_1433dup p.K477dip inframe insertion		BCL6*	CNG		KLHL6*	c.1061C > T p.P354L MV	6.1%
						MSH3	c.316C > G p.Q106E MV		MCL1*	CNG		ERBB4*	c.1618G > T p.D540Y MV	5.7%
									SOX2*	CNG				
	ctDNA		IV	T + 1713		None			None			None		

Colored text: Indicates a mutation observed in one patient’s tissue-based TEMPUS 648 gene panel report and ctDNA-based 105 gene panel report.

*Indicates mutations observed in tissue-based TEMPUS 648 gene panel reports that were not tested for in ctDNA-based TEMPUS 105 gene panel reports.

Abbreviations: CNG, copy number gain; CNL, copy number loss; GOF, gain-of-function; LOF, loss-of-function; MV, missense variant; PM, promoter mutation; SG, stop-gain; SRV, splice region variant; VAF, variant allele frequency.

Table 3 displays the mean number of mutations in tissue and ctDNA, in addition to the percentage of tumor tissue mutations and ctDNA mutations found in ctDNA and tissue, respectively. Among the 11 patients in our study, there were a total of 92 mutations found in tissue and 21 mutations found in ctDNA. The mean number of mutations in tissue was 8.4 (92 mutations/11 patients = 8.4 mutations per patient), and the mean number of mutations in ctDNA was 2.6 (29 mutations/11 patients = 2.6 mutations/patient). The percentage of mutations in ctDNA found in tissue was 34.5% (10 concordant mutations between tissue and ctDNA/29 mutations in ctDNA). Importantly, only 21 of the 92 mutations in tissue were in genes that were also analyzed in the ctDNA-based TEMPUS 105 gene panel reports. Therefore, to calculate the percentage of mutations in tissue found in ctDNA, only mutations in genes that were assessed both by the tissue-based TEMPUS 648 gene panel report and ctDNA-based TEMPUS 105 gene panel report were included. Therefore, the percentage of mutations in tissue found in ctDNA was 47.6% (10 concordant mutations between tissue and ctDNA/21 mutations in tissue).

**Table 3.** Mutations in tumor tissue and ctDNA.

Mean number of mutations in tissue	8.4 mutations per patient
Mean number of mutations in ctDNA	2.6 mutations per patient
Percentage of mutations in ctDNA found in tissue	34.5%
Percentage of mutations in tissue found in ctDNA*	47.6%

Asterisk indicates the exclusion of mutations in genes not tested for by ctDNA-based 105 gene panel reports.

## Discussion

Our analysis outlines the impact of therapy on the mutational concordance between tumor tissue and ctDNA in patients with metastatic SCCA. Importantly, our real-world cohort of patients was similar to other cohorts described in the literature and the US, with regard to rates of HPV-positivity (9 patients; 81.8%) and mutations in *PIK3CA* (5 patients; 45.4%) which is one of the most commonly mutated genes in SCCA.^17,18,24^ The most frequently mutated “somatic—potentially actionable” and “somatic—biologically relevant” genes in tumor tissue among patients in our cohort were *PIK3CA* (4, 5, 9, 10, and 11) and *CYLD* (4, 6, and 7), respectively. Notably, one prior study suggested that mutations in *TP53*, *PIK3CA*, *KMT2C*, *KMT2D*, *RB1*, *FAT4*, *NF1*, *CDKN2A*, and *CTNNB1* are driver mutations of anal cancer development.^25^ We identified several patients with tumor tissue containing mutations in these genes. A *TP53* mutation was found in one patient’s^1^ tumor tissue. *PIK3CA* mutations were found in 5 patients’ (4, 5, 8, 10, and 11) tumor tissue. A *KMT2C* mutation and KMT2D mutation were found in 2 separate patients’ tumor tissues (11 and 8, respectively). *CDKN2A* mutations were found in 2 patients’ (1 and 10) tumor tissue.

### Effect of Therapy on Concordance and VAF

VAF is a surrogate measure of the percentage of DNA in a sample that carries mutations.^26^ A higher VAF, as measured by ctDNA, is a marker of tumor burden and is associated with worse survival.^27^ Notably, genomic alterations with a VAF of less than 1% may not actually represent DNA shed from a patient’s tumor but rather clonal hematopoiesis of indeterminate potential (CHIP).^28^ It has been shown that VAF measured by ctDNA is lower than that of tissue among samples collected before the initiation of therapy in patients with non-small cell lung cancer.^29^ VAF, when measured serially in patients receiving systemic therapy, has been shown to decrease over time in patients who experience a response (determined by imaging) to treatment.^30^ In our analysis, we found that VAF was lower in ctDNA compared to tissue in the majority of concordant mutations (except *PI3KCA* in patient 8 and *RAD51C* and *JAK2* in patient 9). However, it is unclear if this difference was related to the fact that tissue VAF is higher in tissue, generally, or whether this was representative of response to therapy.

### Acquisition of Previously Absent Mutations

When patients with metastatic cancer experience disease progression, it is not uncommon for clinicians to obtain tissue from a new site of disease and assess for emerging actionable mutations with NGS. The viability of utilizing ctDNA for this purpose, as well as to understand whether certain changes in a tumor’s mutational profile are indicative of response (or lack thereof) to therapy, is currently being investigated in several clinical trials in patients with melanoma and NSCLC (NCT04166487, NCT04966676, NCT04093167). Seven (1, 2, 3, 6, 8, 9, and 10) patients had newly identified mutations in ctDNA that were not present in tumor tissue.

Three patients (6, 9, and 10) had potentially actionable mutations in ctDNA that were not previously present in tumor tissue. Patient 6 was found to have a mutation in *FGFR2* (c.1364C>T p.T455M MV; VAF = 49.7%) in ctDNA that was not previously present in tumor tissue, for which several agents have been shown to demonstrate activity in advanced, refractory tumors harboring aberrations in *FGFR 1-3.*^31-33^ This patient was treated with pemigatinib, an FGFR2 inhibitor, and achieved a durable, objective response.^33^ Patient 9 was found to have a mutation in *BRCA1* (c.2029_2030delinsTT p.G677L MV; VAF = 4.8%) in ctDNA that was not previously present in tumor tissue. In a meta-analysis that examined the activity of polyadenosine diphosphate-ribose polymerase (PARP) inhibitors, which have efficacy in specific patient populations with tumors containing *BRCA1/2* mutations, 24 of 43 patients with tumors containing somatic *BRCA* mutations treated with PARP inhibitors had a response to therapy across 8 trials.^34^ Patient 10 was found to have a mutation in *ERBB2* (.234G>T p.Q78H MV; VAF = 28.2%) in ctDNA that was not previously present in tumor tissue. Genetic alterations in *ERBB2* have been established as a biomarker for targeted anti-human epidermal growth factor receptor 2 (HER2) therapy in patients with multiple types of solid tumors, including gastrointestinal malignancies such as gastroesophageal, gastric, and colorectal cancers.^35,36^

Four patients (2, 3, 6, and 8) had mutations with a VAF greater than 45% in ctDNA that were not previously present in tumor tissue, which may indicate they were drivers of resistance to therapy and metastasis. These mutations were in *KDR* (c.1379G>T p.W460L MV; VAF = 50.4%), *SDHA* (c.1663 + 3G>C SRV; VAF = 46.8%), *HNF1A* (c.1336G>A p.V446M MV; VAF = 57.4%), *FGFR2* (c.1364C>T p.T455M MV; VAF = 49.7%), *PBRM1* (c.1409A>G p.Y470C MV; VAF = 85.1%), *DDR2* (c.749T>A p.V250E MV; VAF = 49.9%), and *MYCN* (c.1090C>T p.P364S MV; VAF = 48.6%). The function of the proteins these genes encode varies. According to the National Center for Biotechnology Information’s Gene database, *KDR* encodes vascular endothelial growth factor receptor 2, *SDHA* encodes a major catalytic subunit of a complex in the mitochondrial respiratory chain, *HNF1A* encodes a transcription factor required for the expression of some liver-specific genes, *FGFR2* encodes fibroblast growth receptor 2, *PBRM1* encodes a subunit of a chromatin remodeling complex integral for nuclear hormone-receptor mediated transcriptional activation, *DDR2* encodes a receptor subclass of the receptor tyrosine kinase family, and *MYCN* encodes a nuclear protein involved in the regulation of transcription.^25^

### Limitations

First, our ability to ascertain the true concordance of mutations between tissue and ctDNA was hampered by the fact that only 105 genes were analyzed on the ctDNA-based TEMPUS 105 gene panel reports, while 648 genes were analyzed on the tissue-based TEMPUS 648 gene panel reports. As annotated with a single asterisk (*) in Tables 1 and 2, 10 patients had mutations identified in tissue-based TEMPUS 648 gene panel reports categorized as “somatic—biologically relevant,” and all 11 patients had tissue with mutations categorized as “Variants of unknown significance” that were not tested for in ctDNA-based TEMPUS 105 gene panel reports. It is possible that if ctDNA-based TEMPUS 105 gene panel reports captured mutations in the same 648 genes as the tissue-based TEMPUS 648 gene panel reports, we would have observed a higher mutational concordance between tumor tissue and ctDNA.

Additionally, as seen in the third column of Tables 1 and 2, tumor tissue and ctDNA samples were obtained at separate, discrete points in time. Had our analysis been solely focused on the concordance of tissue and ctDNA mutational signatures rather than also trying to capture the impact of therapy on changes in SCCA mutational signatures, it would have been imperative that both tissue and ctDNA be collected simultaneously either before the start of treatment or at a discrete point in time during the course of therapy.

Lastly, the relatively small number of patients included in our analysis may limit the generalizability of our findings. This highlights the need for multi-institutional registries to study and understand detailed genomic information in uncommon tumor types, such as SCCA.

## Conclusions

Among all solid tumor types, including SCCA, NGS of biopsy-obtained tumor tissue is crucial for identifying genomic alterations that could potentially serve as therapeutic targets. While tissue biopsies have primarily served as the gold standard for identifying mutations in patients with a new cancer diagnosis, noninvasive measures to identify genomic alterations are also being studied as a noninvasive and complementary approach to predict and identify dynamic responses to therapy in the neoadjuvant, adjuvant, and metastatic setting. Our analysis showed a relatively high degree of mutational temporal heterogeneity in patients with SCCA who received locoregional and systemic therapy, supporting the hypothesis that ctDNA may serve as a mechanism to track the real-time evolution of the molecular underpinnings of solid tumors subjected to systemic therapy, which can allow for the identification of mutations that, when present, allow patients with refractory SCCA the opportunity to participate in a clinical trial or receive off-label targeted therapy. As targeted therapies are developed and approved for this rare cancer, the utility of this technique will only become more relevant.

Our study demonstrated some degree of concordance, particularly in commonly mutated genes in SCCA, such as *PI3KCA*, between tumor tissue and ctDNA in patients with SCCA. The absence of evidence for a high mutational concordance between tissue and ctDNA limits the ability to rely solely on ctDNA-based mutations when administering targeted therapy in most tumor types, including SCCA.

Furthermore, integrative models of the tumoral microenvironment and more advanced sequencing technologies of peripheral blood, should continue to be investigated. Another critical area that should be explored is whether dynamic changes in a tumor’s genetic profile, and that of the ctDNA tumor shedding may be correlated to radiologically confirmed response or disease progression and if this information can be used to guide future decisions related to the continuation, de-escalation, or revision of treatment options.

## Data Availability

The data underlying this article are available in the article and in its online supplementary material.

## Prior Presentation

Data from this study was presented at the ASCO 2023 Gastrointestinal Cancer Symposium.

## References

[1] Siegel RL , Miller KD, Fuchs HE, Jemal A. Cancer statistics, 2022. CA Cancer J Clin. 2022;72(1):7-33. 35020204 10.3322/caac.21708

[2] Deshmukh AA , Suk R, Shiels MS, et al. Recent trends in squamous cell carcinoma of the anus incidence and mortality in the united states, 2001-2015. J Natl Cancer Inst. 2020;112(8):829-838. 10.1093/jnci/djz219 31742639 PMC7825484

[3] Daling JR , Madeleine MM, Johnson LG, et al. Human papillomavirus, smoking, and sexual practices in the etiology of anal cancer. Cancer. 2004;101(2):270-280. 15241823 10.1002/cncr.20365

[4] Johnson LG , Madeleine MM, Newcomer LM, Schwartz SM, Daling JR. Anal cancer incidence and survival: the surveilance, epidemiology, and end results experience, 1973-2000. Cancer. 2004;101(2):281-288. 10.1002/cncr.20364 15241824

[5] Baricevic I , He X, Chakrabarty B, et al. High-sensitivity human papilloma virus genotyping reveals near universal positivity in anal squamous cell carcinoma: different implications for vaccine prevention and prognosis. Eur J Cancer. 2015;51(6):776-785. 10.1016/j.ejca.2015.01.058 25702585

[6] Ajani JA , Winter KA, Gunderson LL, et al. Fluorouracil, mitomycin, and radiotherapy vs fluorouracil, cisplatin, and radiotherapy for carcinoma of the anal canal: a randomized controlled trial. JAMA. 2008;299(16):1914-1921. 10.1001/jama.299.16.1914 18430910

[7] James RD , Glynne-Jones R, Meadows HM, et al. Mitomycin or cisplatin chemoradiation with or without maintenance chemotherapy for treatment of squamous-cell carcinoma of the anus (ACT II): a randomised, phase 3, open-label, 2 × 2 factorial trial. Lancet Oncol. 2013;14(6):516-524. 10.1016/S1470-2045(13)70086-X 23578724

[8] Eng C , Chang GJ, You YN, et al. The role of systemic chemotherapy and multidisciplinary management in improving the overall survival of patients with metastatic squamous cell carcinoma of the anal canal. Oncotarget. 2014;5(22):11133-11142. 10.18632/oncotarget.2563 25373735 PMC4294384

[9] Rao S , Sclafani F, Eng C, et al. International rare cancers initiative multicenter randomized phase II trial of cisplatin and fluorouracil versus carboplatin and paclitaxel in advanced anal cancer: InterAAct. J Clin Oncol. 2020;38(22):2510-2518. 32530769 10.1200/JCO.19.03266 PMC7406334

[10] Thind G , Johal B, Follwell M, Kennecke HF. Chemoradiation with capecitabine and mitomycin-C for stage I-III anal squamous cell carcinoma. Radiat Oncol. 2014;9(1):124. 10.1186/1748-717X-9-124 24885554 PMC4050390

[11] Roth MT , Catalano PJ, Ciombor KK, et al. A randomized phase III study of immune checkpoint inhibition with chemotherapy in treatment-naive metastatic anal cancer patients: a trial of the ECOG-ACRIN cancer research group (EA2176). J Clin Oncol. 2021;39(15_suppl):TPS3614.

[12] Ott PA , Piha-Paul SA, Munster P, et al. Safety and antitumor activity of the anti-PD-1 antibody pembrolizumab in patients with recurrent carcinoma of the anal canal. Ann Oncol. 2017;28(5):1036-1041. 10.1093/annonc/mdx029 28453692 PMC5406758

[13] Alvarez J , Cercek A, Mohan N, et al. Circulating tumor DNA (ctDNA) to assess response in patients with anal cancer treated with definitive chemoradiation. [Internet]. J Clin Oncol. 2023;41(16_suppl). https://ascopubs.org/doi/abs/10.1200/JCO.2023.41.4_suppl.1

[14] Cabel L , Jeannot E, Bieche I, et al. Prognostic impact of residual HPV ctDNA detection after chemoradiotherapy for anal squamous cell carcinoma. Clin Cancer Res. 2018;24(22):5767-5771. 10.1158/1078-0432.CCR-18-0922 30054279

[15] Gerlinger M , Rowan AJ, Horswell S, et al. Intratumor heterogeneity and branched evolution revealed by multiregion sequencing. N Engl J Med. 2012;366(10):883-892. 22397650 10.1056/NEJMoa1113205 PMC4878653

[16] Hiley C , de Bruin EC, McGranahan N, Swanton C. Deciphering intratumor heterogeneity and temporal acquisition of driver events to refine precision medicine. Genome Biol. 2014;15(8):453. 25222836 10.1186/s13059-014-0453-8 PMC4281956

[17] Chung JH , Sanford E, Johnson A, et al. Comprehensive genomic profiling of anal squamous cell carcinoma reveals distinct genomically defined classes. Ann Oncol. 2016;27(7):1336-1341. 10.1093/annonc/mdw152 27052656

[18] Morris V , Rao X, Pickering C, et al. Comprehensive genomic profiling of metastatic squamous cell carcinoma of the anal canal. Mol Cancer Res. 2017;15(11):1542-1550. 10.1158/1541-7786.MCR-17-0060 28784613 PMC5991496

[19] Chae YK , Davis AA, Carneiro BA, et al. Concordance between genomic alterations assessed by next-generation sequencing in tumor tissue or circulating cell-free DNA. Oncotarget. 2016;7(40):65364-65373. 27588476 10.18632/oncotarget.11692 PMC5323161

[20] Chae YK , Davis AA, Jain S, et al. Concordance of genomic alterations by next-generation sequencing in tumor tissue versus circulating tumor DNA in breast cancer. Mol Cancer Ther. 2017;16(7):1412-1420. 28446639 10.1158/1535-7163.MCT-17-0061

[21] Imperial R , Nazer M, Ahmed Z, et al. Matched whole-genome sequencing (WGS) and whole-exome sequencing (WES) of tumor tissue with circulating tumor DNA (ctDNA) analysis: complementary modalities in clinical practice. Cancers (Basel). 2019;11(9):1399. 10.3390/cancers11091399 31546879 PMC6770276

[22] Park S , Olsen S, Ku BM, et al. High concordance of actionable genomic alterations identified between circulating tumor DNA–based and tissue-based next-generation sequencing testing in advanced non–small cell lung cancer: The Korean Lung Liquid Versus Invasive Biopsy Program. Cancer. 2021;127(16):3019-3028. 33826761 10.1002/cncr.33571

[23] Chang YS , Fang HY, Hung YC, et al. Correlation of genomic alterations between tumor tissue and circulating tumor DNA by next-generation sequencing. J Cancer Res Clin Oncol. 2018;144(11):2167-2175. 10.1007/s00432-018-2747-9 30203147 PMC11813330

[24] Smaglo BG , Tesfaye A, Halfdanarson TR, et al. Comprehensive multiplatform biomarker analysis of 199 anal squamous cell carcinomas. Oncotarget. 2015;6(41):43594-43604. 10.18632/oncotarget.6202 26498363 PMC4791253

[25] Martínez-Jiménez F , Muiños F, Sentís I, et al. A compendium of mutational cancer driver genes. Nat Rev Cancer. 2020;20(10):555-572. 32778778 10.1038/s41568-020-0290-x

[26] Strom SP. Current practices and guidelines for clinical next-generation sequencing oncology testing. Cancer Biol Med. 2016;13(1):3-11. 27144058 10.28092/j.issn.2095-3941.2016.0004 PMC4850126

[27] Pairawan S , Hess KR, Janku F, et al. Cell-free circulating tumor DNA variant allele frequency associates with survival in metastatic cancer. Clin Cancer Res. 2020;26(8):1924-1931. 31852833 10.1158/1078-0432.CCR-19-0306 PMC7771658

[28] Jaiswal S , Fontanillas P, Flannick J, et al. Age-related clonal hematopoiesis associated with adverse outcomes. N Engl J Med. 2014;371(26):2488-2498. 10.1056/NEJMoa1408617 25426837 PMC4306669

[29] Ettrich TJ , Schwerdel D, Dolnik A, et al. Genotyping of circulating tumor DNA in cholangiocarcinoma reveals diagnostic and prognostic information. Sci Rep. 2019;9(1):13261. 10.1038/s41598-019-49860-0 31519967 PMC6744511

[30] Tran HT , Lam VK, Elamin YY, et al. Clinical outcomes in non–small-cell lung cancer patients treated with EGFR-tyrosine kinase inhibitors and other targeted therapies based on tumor versus plasma genomic profiling. JCO Precis Oncol. 2021;5(5):1241-1249. 10.1200/PO.20.00532 PMC8345916 34377884

[31] Chae YK , Hong F, Vaklavas C, et al. Phase II study of AZD4547 in patients with tumors harboring aberrations in the FGFR pathway: results from the NCI-MATCH Trial (EAY131) subprotocol W. J Clin Oncol. 2020;38(21):2407-2417. 10.1200/JCO.19.02630 32463741 PMC7367548

[32] Bahleda R , Meric-Bernstam F, Goyal L, et al. Phase I, first-in-human study of futibatinib, a highly selective, irreversible FGFR1–4 inhibitor in patients with advanced solid tumors. Ann Oncol. 2020;31(10):1405-1412. 32622884 10.1016/j.annonc.2020.06.018 PMC9743148

[33] Miranda KW , Cimino SK, Eng C. Targeted fibroblast growth factor receptor (FGFR) inhibition in recurrent, metastatic anal carcinoma: a case report. Clin Colorectal Cancer. 2022;21(3):272-275. 10.1016/j.clcc.2022.01.001 35125320

[34] Mohyuddin GR , Aziz M, Britt A, et al. Similar response rates and survival with PARP inhibitors for patients with solid tumors harboring somatic versus Germline BRCA mutations: a Meta-analysis and systematic review. BMC Cancer. 2020;20(1):507. 10.1186/s12885-020-06948-5 32493233 PMC7267765

[35] Strickler JH , Ng K, Cercek A, et al. MOUNTAINEER:open-label, phase II study of tucatinib combined with trastuzumab for HER2-positive metastatic colorectal cancer (SGNTUC-017, trial in progress). J Clin Oncol. 2021;39(3_suppl).

[36] Bang YJ , van Cutsem E, Feyereislova A, et al; ToGA Trial Investigators. Trastuzumab in combination with chemotherapy versus chemotherapy alone for treatment of HER2-positive advanced gastric or gastro-oesophageal junction cancer (ToGA): a phase 3, open-label, randomised controlled trial. Lancet. 2010;376(9742):687-697. 10.1016/S0140-6736(10)61121-X 20728210

